# Exploring how GPs discuss statin deprescribing with older people: a qualitative study

**DOI:** 10.3399/bjgpopen20X101022

**Published:** 2020-04-01

**Authors:** Wade Thompson, Jette Videbæk Le, Peter Haastrup, Jesper Bo Nielsen, Line Bjørnskov Pedersen, Dorte Ejg Jarbøl

**Affiliations:** 1 PhD Student, Research Unit of General Practice, University of Southern Denmark, Odense, Denmark; 2 PhD Student, Hospital Pharmacy Funen, Odense University Hospital, Odense, Denmark; 3 Visiting Researcher, Research Unit of General Practice, University of Southern Denmark, Odense, Denmark; 4 Associate Professor, Research Unit of General Practice, University of Southern Denmark, Odense, Denmark; 5 Professor, Research Unit of General Practice, University of Southern Denmark, Odense, Denmark; 6 Associate Professor, Danish Centre for Health Economics, University of Southern Denmark, Odense, Denmark; 7 Senior Researcher, Research Unit of General Practice, University of Southern Denmark, Odense, Denmark; 8 Professor, Research Unit of General Practice, University of Southern Denmark, Odense, Denmark

**Keywords:** hydroxymethylglutaryl-CoA reductase inhibitors, deprescribing, decision making, shared, general practice

## Abstract

**Background:**

Given uncertainty surrounding benefits and harms, shifts in patient health status, and changing patient goals and preferences, statin deprescribing may be considered in some older people. This decision should be carefully discussed between GPs and patients.

**Aim:**

To explore how GPs discuss deprescribing of statins with their older patients.

**Design & setting:**

A qualitative study was undertaken using face-to-face, semi-structured interviews with Danish GPs from the regions of Southern Demark and Zealand.

**Method:**

The GP participants belonged to group practices and were identified from personal networks and snowballing. The interviews lasted approximately 30 minutes and were conducted in English. They were analysed using systematic text condensation.

**Results:**

A total of 11 GPs were interviewed and three themes were identified. (1) Reason for initiating a discussion: statin deprescribing mainly came up when GPs reviewed medication lists. There were differences between GPs regarding when or if they brought up deprescribing. (2) Discussion topics: GPs often discussed their interpretation of evidence surrounding statin use in older people. There were differences in how and if GPs discussed patient preferences. GPs viewed uncertainty and life expectancy as difficult to discuss. (3) Depth of discussion: the perceived level of patient engagement, and clinical context, could influence the extent of discussion.

**Conclusion:**

GPs identified a range of topics that could be discussed with patients surrounding statin deprescribing. The depth and content of discussions varied according to the situation, and between GPs. Challenges may exist in communicating around certain topics, such as uncertainty and life expectancy. Further understanding of how to best communicate around challenging topics, and development of structured frameworks, may help facilitate statin deprescribing discussions. Identifying what patients think is important to discuss would provide necessary insight to promote quality discussions and shared understanding of the decision.

## How this fits in

Shifting treatment goals, uncertainty surrounding ongoing benefit, and increased potential for medication-related harm, mean that some preventive medications may no longer be a good fit for people as they age. Decisions surrounding ongoing use of preventive medications, including statins, should be individualised. Individualised decision-making should involve a careful discussion between patients and GPs. Earlier studies show that patients are open to discussing ongoing statin use, but there is little understanding of how discussions currently take place (for example, what topics are discussed and how). This study sheds light on what Danish GPs are currently discussing with older people surrounding statin deprescribing. It also suggests which topics are more challenging to discuss than others in the context of deprescribing. This suggests the possible need for structured frameworks to both facilitate statin deprescribing discussions and promote shared understanding of the decision.

## Introduction

Statins are commonly used in older people.^1^ While statins may have been initiated with a compelling and evidence-based indication, the balance of benefit and harm of statins can shift as people age,^2^ just as patients’ goals of care and treatment preferences can also change.^3^ The evidence for the benefit of statin treatment is uncertain in people aged >80 years (defined as the ‘oldest old’ in this study), particularly for primary prevention and for those who are frail or have multimorbidity.^2,4^ As such, long-term statin treatment should be reassessed to ensure it continues to be the best choice for people as they age.^4,5^ This has been acknowledged, to varying degrees, in guidelines and expert opinion on cardiovascular (CV) risk reduction, including Danish guidelines, which generally advocate for an individualised approach to statin use in people aged >80 years.^4–6^ As part of this individualised approach, deprescribing (planned, supervised discontinuation)^4,5,7^ of a statin may be considered. Therefore, patients and prescribers may be faced with the decision of continuation versus deprescribing of a statin. Established decision-making frameworks^8–10^ suggest that medication decisions be informed; consistent with patient values, preferences, and goals; and shared between the patient and clinician.^11^ Patients and prescribers should have a common understanding of options that exist; knowledge of benefits, harms, and uncertainties for options; and values and preferences of the patient.^9,10,12^ As such, medication decisions, including statin deprescribing, require careful discussion between patients and prescribers around these various considerations. Patients appear to be willing to consider the option of deprescribing when it is brought forward by their doctor.^13^ However, discussing medication deprescribing can be challenging for GPs in clinical practice.^14–16^ This may, in part, be owing to limited evidence on deprescribing, time and workflow constraints, and perceived self-efficacy.^14,15^ A study on proton pump inhibitors and benzodiazepine deprescribing discussions^17^ suggests that the structure and content of discussions may depend on the medication and patient context. While GPs’ approach to decisions around deprescribing CV preventive medication decisions^18,19^ has been investigated, there has been little exploration into how GPs actually discuss potential statin deprescribing, specifically among older people. As such, the study aimed to explore how GPs discuss statin deprescribing in their older patients.

## Method

A qualitative study was conducted, consisting of face-to-face, semi-structured interviews with GPs.

### ​Participants

GPs were from the regions of Southern Denmark and Zealand. Participants were identified from personal networks and snowballing. It was asked that participants had self-assessed experience discussing long-term use of statins with their oldest old patients. The aim was to recruit GPs with different ages, years of practice, location (urban versus rural), and sex. The study continued to recruit GPs until sufficient data had been obtained to answer the research question.^20^

### ​Interview guide

The interview guide was drafted from existing literature on shared decision-making,^8–10^ GP attitudes towards medication discontinuation,^15^ and based on clinical experience of the research group. It centred on identifying how GPs discuss potential statin deprescribing in their oldest old (aged >80 years) patients. The interview guide was developed by a research team consisting of physicians with experience in general practice, a pharmacist, and researchers with experience in qualitative research and risk communication. The guide was updated based on iterative reviews by the author team. The interview guide was piloted with two GPs and the guide was revised based on these pilot interviews. Please see Supplementary Appendix S1 for the full interview guide.

### ​Interviews and analysis

Interviews lasted approximately 30 minutes and were conducted in English. The interviews were audio-recorded and transcribed verbatim. GPs were de-identified and assigned a number. Analysis was conducted based on systematic text condensation.^21^ First, two researchers read through all interviews, while the other researchers read through two to three interviews each. The group met to discuss and agree on preliminary themes. Once preliminary themes were defined, one researcher identified meaning units (quotations or text fragments from the transcripts that represented aspects of the themes) from all interviews. The meaning units were then grouped according to which preliminary theme they belonged to. With all meaning units grouped together, the themes that were initially developed were refined. Themes were refined based on team discussion and meaning units could be reorganised under themes as necessary. This flexibility is in keeping with systematic text condensation methods. Sub-themes were then established within each main theme, and meaning units were organised under each sub-theme. A condensate was created from all meaning units in a sub-theme. A condensate is a coherent artificial quotation that captures all the meaning units within a sub-theme. The condensates and meaning units were used to develop an analytic text (a presentation of the content and meaning of the sub-theme) for each sub-theme. Analytic texts were used as the results section.

## Results

**Figure 1.**
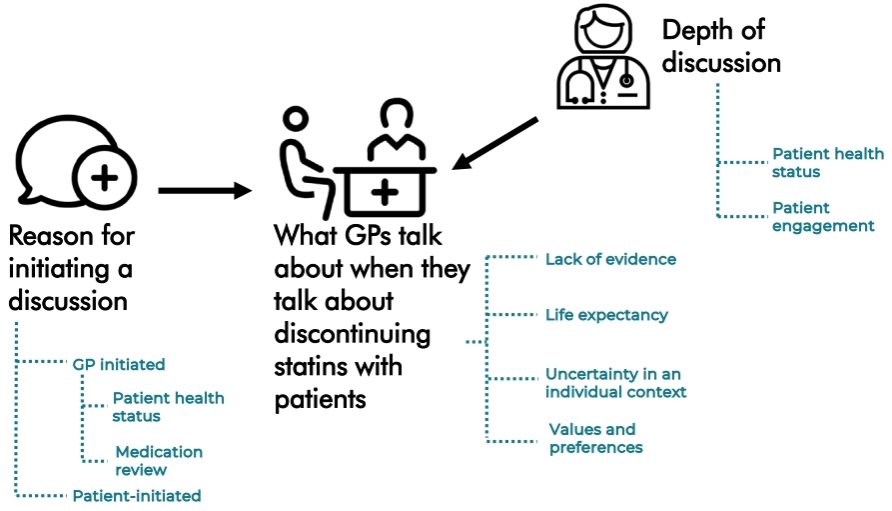
Themes and sub-themes. New Conversation icon by Gregor Cresnar, Doctor Consultation icon by Delwar Hossain, Doctor icon by Aficons; all icons courtesy of thenounproject.com.

A total of 11 GPs were recruited. The characteristics of the GPs are in Table 1. All GPs belonged to group practices. Three themes were identified related to discussions surrounding statin deprescribing, which are summarised below and in Figure 1.

**Table 1.** GP characteristics

**Characteristic**	(*n* = 11)
Mean age (SD, range)	46 (7.9) [41–68]
Years practising (mean, SD, range)	9 (7.6) [3–30]
Practice location (*n*, %)	
Rural	2 (18)
Urban	9 (82)
Biological sex (*n*, %)	
Female	6 (55)
Male	5 (45)

SD = standard deviation

### Theme 1: Reason for initiating a discussion

The interviews revealed two main reasons a deprescribing discussion could come up: (1) patients initiating the discussion; or (2) GPs initiating the discussion when reviewing medication lists (based on a GP’s assessment of patient health status).

#### ​Patients initiating the discussion

Patients could sometimes bring up statin discontinuation. Most GPs reported that if a patient wanted to discuss statins, it was because they were worried about adverse effects, although patients or caregivers also brought up possible discontinuation owing to concerns about taking too much medication (in this context, statins would not be brought up specifically but as part of a strategy of reducing medications in general):

*'Many people think they have side effects from statins. So they always bring this subject up. They say … “I have some pain in my legs, could it be because of the statins?”'* (GP 4)

*​'*[Patients] *say “doctor I take so much medicine, couldn’t I remove some of it?” We go, yeah what do you need? What is really necessary? And what could we try to stop?'* (GP 9)

#### ​GPs initiating the discussion during a medication review

The most common way a discussion would be started was if the GP brought it up when they were reviewing a patient’s medication list, for example, during an annual medical review or nursing home admission. Statins were often not a specific focus but came up together in a discussion of all the patient’s medications:

*'I had a lot of yearly consultations today ... usually at these annual meetings, I tell* [patients] *that it’s very important that we go through their medication list to check what they get, what the reason is, and why we sometimes might need to change it or stop.'* (GP 10)

#### *​*GPs initiating the discussion based on patient health status

GPs noted that they would not bring up deprescribing in all older people taking statins. The GP’s assessment of the patient’s health status appeared to influence whether a discussion was initiated. Most GPs stated that if a patient appeared fit and healthy, they would not initiate a discussion around ongoing statin use:

*​'If you are 80 and healthy and* [have] *no side effects and you are playing football … and biking, then I wouldn’t remove it.'* (GP 9)

Conversely, GPs described factors that could lead them to initiate a discussion on statin deprescribing. Different GPs mentioned different factors, such as whether a patient had trouble swallowing pills, took a lot of medication, or had a lot of health complaints. Some GPs also considered CV risk, while one GP noted that they would not bring up statin discontinuation until end of life:

*​ 'If they don’t feel good. If they are tired, or if they have a pain problem. Or if there’s something with their appetite. Then I would consider* [discontinuation] *more.'* (GP 8)

*​'And whenever they get into sort of the end stage of dementia or the end stage of whatever illness, we remove the statins.'* (GP 7)

*​* *'* *… it really depends on who they are and what have they been through. Do they have diabetes and hypertension and have had one, two, three strokes before?* *'* (GP 6)

### Theme 2: What GPs talk about when they talk about statin discontinuation with patients

GPs described four topics that could be discussed: limited evidence for benefit, life expectancy, uncertainty in an individual context, and patient goals and preferences.

#### Lack of evidence

One possible discussion point most GPs mentioned was their interpretation of the evidence for benefit of statins in the oldest old. Most GPs said that they would talk about how statins have not been well-studied in people aged 80 or 90 years:

*​'I would say: … But what we don’t know is that now you have come into your 80s , no one has really examined if it has any meaning to have the medication in this period.'* (GP 2)

GPs may also mention that possible benefits were seen over a long period. This could be discussed in general terms, although some GPs would talk about a specific timeframe (for example, 10 years).

GPs also described situations where they would simply tell patients they were unlikely to benefit from a statin, such as in patients who were at the end of life or very unwell:

*​'It could be a patient with COPD or a patient that was in a bad way, then I would probably just tell them that the risk of side effects is probably larger than the chance of benefitting from the medication.'* (GP 5)

#### ​Life expectancy

There were differing views on discussing life expectancy. Most GPs thought about life expectancy, but some did not necessarily bring it up directly, as they viewed this as being a difficult topic to discuss. Some GPs said they would discuss it indirectly. For example, one GP said that if they mentioned that statins were used to reduce long-term risk, then people would understand. Another GP said that they felt the idea of life expectancy was unsaid but understood:

*​'I mention that it is a prophylactic treatment, that maybe you can benefit* [from] *it in 10–15 years. Most of the patients laugh a little when I say that. “Oh, it’s 15 years. That long … that’s OK, we can stop it.” Of course, I don’t mention they only have so long to live.'* (GP 5)

In contrast, one GP felt that length of life was a natural topic when discussing statins:

*​ 'Of course we talk about the length of life and the quality of life, and I think that should be very natural.'* (GP 10)

#### ​Uncertainty in an individual context

Uncertainty in an individual context was also a possible discussion point. Some GPs would discuss the challenge of knowing for certain whether an individual person would benefit from a statin (or avoid a stroke or heart attack if discontinuing, for example). GPs noted this as a difficult topic since patients want the GPs to be sure about the course of action:

*​'I think for many people it's very hard to have this discussion.* [Patients] *really want us to be sure. Are you sure I won't get a stroke if I continue. Now are you sure that I will get one if I stop? No.'* (GP 6)

*​'It's one of the most difficult questions to discuss with patients and their families because it’s so abstract. It is very difficult to explain ... I can't point to your mum and say it will benefit or harm her.'* (GP 11)

#### ​Patient goals and preferences

GPs acknowledged patient preferences and goals as important. Different GPs described different ways goals and preferences could be brought up and different topics that could be discussed. Some GPs talked about preferences towards medication use, while some also discussed the issue of prolonging life versus quality of life:

*​'That is something we talk about very often. I ask the family or the patient ... what do you think about? ... What is important in your life right now? Is it … life quality without taking too much medicine and potentially getting side effects and so on?'* (GP 8)

While some GPs openly discussed goals and preferences, others spoke of a general awareness of people's goals and preferences, feeling they knew a person’s preferences and goals without asking:

*​ 'Yeah it’s understood what the goals are. I can feel that the patient is tired of living.'* (GP 4)

### Theme 3: Depth of discussion

Two factors were important to GPs when determining the depth of the discussion: the patient’s health status; and the perceived level of patient engagement.

#### ​Patient health status

GPs talked about how patient health status could affect the depth of the discussion. Here, they mentioned factors such as frailty, life expectancy, and dementia status. GPs described situations where they would typically have minimal discussion; for example, in those at the end of life or those the GPs described as very sick:

*​'At that level we basically don't discuss it with the patient. Or the family. We simply say now we are reducing the medication because now they are approaching the end of life.'* (GP 7)

*​'If they’re getting a lot of care I would probably not have that much of a discussion. I would probably just tell the patient.'* (GP 5)

#### ​Patient engagement

GPs discussed their perception of a person’s willingness to engage in a discussion. Some GPs felt certain older patients would prefer the decision be left up to the GP. In such patients, there would be minimal discussion:

*​'I mean some of my older patients just say you choose, and I will do it.'* (GP 10)

GPs also explained how the discussion was affected by whether patients had read a lot of information on the internet or had engaged family members who were keen to discuss the subject:

*'Other patients have read everything on the internet ... Then of course it’s very much a longer discussion.'* (GP 10)

Some GPs suggested they would try to have a more open discussion regardless of the perceived level of engagement:

*​'I like to look everything up with the patient because I like that our knowledge is the same, and they know my doubts and thoughts. If they have a high risk, then they know they have a high risk and if they have a low risk I want them to know it. So we can share the risk and so I can meet them in a place where we are in an understanding of why they should get the medication. But if I just tell them what to do it is not a good way of working together. Every consultation is a shared agreement.'* (GP 2)

## Discussion

### ​Summary

The GPs that were interviewed were aware of the clinical heterogeneity in older people. Therefore, they generally endorsed individualising whether to bring up statin deprescribing, and the discussion itself, depending on patient-specific factors and the clinical situation. GPs described different topics they might discuss with patients. Some topics were seen as being more difficult to discuss compared with others, such as life expectancy and uncertainty with respect to statin deprescribing.

### ​Strengths and limitations

GPs were recruited through personal networks of the researchers. Participating GPs may have been those who are more open to the idea of discussing statin deprescribing than the general population of Danish GPs. GPs were only recruited from two of the five regions in Denmark, but substantial diversity between regions was not expected. GPs from both single-handed and partnership practices were sought for participation, but owing to slow recruiting, the study only recruited GPs in partnership practices. However, informants did have a range of ages and practice experience as well as a roughly equal sex distribution. Another possible limitation is that interviews were conducted in English. This could have affected how GPs expressed themselves, but the level of proficiency in English is high among Danish people,^22^ and GPs in the interviews had the opportunity to provide answers in Danish if necessary. Thus, it is not expected that language had an impact on the findings. Desirability bias is possible, where GPs wanted to provide answers that reflect their perception of an optimal approach to discussing statin deprescribing. However, it was clearly outlined that there were no right answers or approaches at the beginning of the interview in an attempt to mitigate this bias. Further, interviews were prefaced by asking informants to base answers on specific instances where they had actually discussed statin deprescribing.

### ​Comparison with existing literature

The GPs interviewed in this study endorsed considering patient-specific factors when bringing up deprescribing. This is consistent with survey data^18^ and previous qualitative work,^19^ suggesting GPs weigh patient factors (for example, CV risk, frailty, and life expectancy) when considering deprescribing of statins. Like Jansen *et al*,^19^ it was noted that there were some differences in how GPs weighed patient-specific factors when bringing forward possible deprescribing.

The interviews suggested that GPs individualise how and what to discuss with patients. For example, they may have minimal discussion with people whom they deem very sick, and more detailed discussions with patients who were very engaged. The extent to which this approach is amenable to patients is unclear. Shared decision-making is acknowledged as an optimal approach to deprescribing.^23^ Yet, interviews with older people in Australia suggest a range of preferences towards actual involvement in shared decision-making surrounding deprescribing.^24^ The authors of this study suggest a typology of patients preferring to defer decision-making to a prescriber, whereas other patients may prefer more involvement. A focus group study in Canada also suggested that desired involvement in decision-making may be a continuum.^25^ Thus, it is possible that a more in-depth discussion may be reasonable to some, where minimal discussion would be satisfactory to other patients. However, it has also been suggested that shared decision-making can still be implemented even when patients desire limited involvement in the decision itself.^26,27^ Patients can still deliberate around the decision, including discussing their goals and preferences and their thoughts on uncertainty, even if they prefer the final decision to be made by the prescriber.^27^ Eliciting goals and preferences is a central component of shared decision-making,^9^ and is likely particularly important in decisions surrounding deprescribing of statins.^23^ Patient attitudes and preferences towards deprescribing of CV medications appears to vary.^28^ This supports the idea that discussing these considerations is important regardless of a patient’s desired level of involvement in a final decision. GPs in this study acknowledged patient values and preferences as important, although there were differences among GPs in how they were discussed, and whether they were discussed.

When entering a full discussion, GPs generally focused on discussing the rationale for considering deprescribing a statin (the question of 'if' deprescribing should be pursued).^17^ This primarily included the GP discussing their interpretation of statin evidence in the oldest old. This is encouraging, as understanding the rationale for a deprescribing option appears to be important to patients.^29^ Some GPs noted that they had difficulty bringing life expectancy up because they felt patients would not want to discuss it. It appears some patients do not wish to discuss life expectancy, with a survey of older people in the US finding that around half of older adults prefer not to discuss life expectancy openly.^30^ This presents a challenge to both GPs and patients in having a full, open discussion surrounding statin use, since life expectancy may be an important consideration in weighing the option of deprescribing. Uncertainty was also seen as being difficult to discuss. GPs in Australia,^14^ the Netherlands,^31^ and New Zealand^16^ have also noted that uncertainty makes deprescribing decisions difficult. However, awareness and discussion of such topics is likely an important component of a shared decision surrounding deprescribing statins, given the current evidence base. Difficulty in discussing uncertainty may be a barrier to employing shared decisions surrounding deprescribing in practice.

### ​Implications for research and practice

The findings point to areas of future research related to communication around deprescribing statins. First, it would be helpful to understand the views and practices of a larger, more representative group of GPs using a quantitative method, such as a survey. Such work could, for example, quantify the relative importance of discussion topics or investigate possible differences in preferences between groups of GPs. The challenges the informants mentioned in communicating topics, such as uncertainty and life expectancy, suggest there may be a need to identify the optimal ways to communicate (or bring up) these topics in relation to deprescribing. Further, the interviews point to the need for further investigation around optimal ways to elicit goals, values, and preferences, in the context of deprescribing discussions. Such studies would clearly incorporate research on patient views towards discussing statin deprescribing. Since a tailored approach to discussions may be appropriate,^17,24^ further understanding of how patients would like to discuss deprescribing will also improve knowledge around how to best tailor discussions (for example, investigating the extent to which patients would like to deliberate around decisions).

Given possible differences in how GPs discuss statin deprescribing and challenges in discussing certain topics, development of more structured frameworks towards having discussions would likely be helpful, ensuring that discussions allow for shared decisions and coverage of topics considered important to both patients and GPs. The need for tools to elicit values and preferences in the context of deprescribing has been suggested following a recent Canadian focus group study.^25^ A framework towards statin deprescribing decisions has also recently been proposed.^4^ While not focused specifically on how to discuss statin deprescribing, it may serve as a useful starting point surrounding possible topics. Knowledge around how GPs discuss (and would like to discuss) statin deprescribing will also be helpful to highlight opportunities for training and development of clinical practice tools. All such work would aim to promote shared decisions surrounding statin deprescribing in clinical practice.

## References

[1] Thompson W, Pottegård A, Nielsen JB (2018). How common is statin use in the oldest old?. Drugs Aging.

[2] van Middelaar T, Moll van Charante EP (2018). Deprescribing preventive medication in older patients. Br J Gen Pract.

[3] Boyd C, Smith CD, Masoudi FA (2019). Decision making for older adults with multiple chronic conditions: Executive summary for the American geriatrics Society guiding principles on the care of older adults with multimorbidity. J Am Geriatr Soc.

[4] Hawley CE, Roefaro J, Forman DE, Orkaby AR (2019). Statins for primary prevention in those aged 70 years and older: a critical review of recent cholesterol guidelines. Drugs Aging.

[5] Krishnaswami A, Steinman MA, Goyal P (2019). Deprescribing in older adults with cardiovascular disease. J Am Coll Cardiol.

[6] Strandberg TE, Kolehmainen L, Vuorio A (2014). Evaluation and treatment of older patients with hypercholesterolemia: a clinical review. JAMA.

[7] Reeve E, Gnjidic D, Long J, Hilmer S (2015). A systematic review of the emerging deﬁnition of 'deprescribing' with network analysis: implications for future research and clinical practice. Br J Clin Pharmacol.

[8] Elwyn G, Durand MA, Song J (2017). A three-talk model for shared decision making: multistage consultation process. BMJ.

[9] Elwyn G, Cochran N, Pignone M (2017). Shared decision Making — the importance of diagnosing preferences. JAMA Intern Med.

[10] Lehman R (2017). Sharing as the future of medicine. JAMA Intern Med.

[11] Hoffmann T, Jansen J, Glasziou P (2018). The importance and challenges of shared decision making in older people with multimorbidity. PLoS Med.

[12] Staveley I, Sullivan P (2015). We need more guidance on shared decision making. Br J Gen Pract.

[13] Qi K, Reeve E, Hilmer SN (2015). Older people's attitudes regarding polypharmacy, statin use and willingness to have statins deprescribed in Australia. Int J Clin Pharm.

[14] Anderson K, Foster M, Freeman C (2017). Negotiating 'unmeasurable harm and benefit': perspectives of general practitioners and consultant pharmacists on deprescribing in the primary care setting. Qual Health Res.

[15] Anderson K, Stowasser D, Freeman C, Scott I (2014). Prescriber barriers and enablers to minimising potentially inappropriate medications in adults: a systematic review and thematic synthesis. BMJ Open.

[16] Ailabouni NJ, Nishtala PS, Mangin D, Tordoff JM (2016). Challenges and enablers of deprescribing: a general practitioner perspective. PLoS One.

[17] Turner JP, Richard C, Lussier M-T (2018). Deprescribing conversations: a closer look at prescriber–patient communication. Ther Adv Drug Saf.

[18] van der Ploeg MA, Streit S, Achterberg WP (2019). Patient characteristics and general practitioners' advice to stop statins in oldest-old patients: a survey study across 30 countries. J Gen Intern Med.

[19] Jansen J, McKinn S, Bonner C (2017). General practitioners' decision making about primary prevention of cardiovascular disease in older adults: a qualitative study. PLoS One.

[20] Malterud K, Siersma VD, Guassora AD (2016). Sample size in qualitative interview studies: guided by information power. Qual Health Res.

[21] Malterud K (2012). Systematic text condensation: a strategy for qualitative analysis. Scand J Public Health.

[22] Education First (2019). English Proficiency Index: 2019 rankings. https://www.ef.com/__/~/media/centralefcom/epi/downloads/full-reports/v9/ef-epi-2019-english.pdf.

[23] Jansen J, Naganathan V, Carter SM (2016). Too much medicine in older people? deprescribing through shared decision making. BMJ.

[24] Weir K, Nickel B, Naganathan V (2018). Decision-Making preferences and deprescribing: perspectives of older adults and companions about their medicines. J Gerontol B Psychol Sci Soc Sci.

[25] Mangin D, Risdon C, Lamarche L (2019). 'I think this medicine actually killed my wife': patient and family perspectives on shared decision-making to optimize medications and safety. Ther Adv Drug Saf.

[26] Légaré F, Thompson-Leduc P (2014). Twelve myths about shared decision making. Patient Educ Couns.

[27] Politi MC, Dizon DS, Frosch DL (2013). Importance of clarifying patients' desired role in shared decision making to match their level of engagement with their preferences. BMJ.

[28] Luymes CH, Boelhouwer NJ, Poortvliet RK (2017). Understanding deprescribing of preventive cardiovascular medication: a Q-methodology study in patients. Patient Prefer Adherence.

[29] Reeve E, To J, Hendrix I (2013). Patient barriers to and enablers of deprescribing: a systematic review. Drugs Aging.

[30] Schoenborn NL, Janssen EM, Boyd C (2018). Older adults' preferences for discussing long-term life expectancy: results from a national survey. Ann Fam Med.

[31] van Middelaar T, Ivens SD, van Peet PG (2018). Prescribing and deprescribing antihypertensive medication in older people by Dutch general practitioners: a qualitative study. BMJ Open.

